# The Anti-Depression-Like Effects of Zhengtian Capsule *via* Induction of Neurogenesis and the Neurotrophic Signaling Pathway

**DOI:** 10.3389/fphar.2020.01338

**Published:** 2020-08-26

**Authors:** Liang Yang, Yong Wang, Nuomin Li, Bing Xu, Juanhui Duan, Chunxu Yuan, Qinfen Yuan, Qifan Yang, Hong Qing, Zhi Dai, Zhenzhen Quan

**Affiliations:** ^1^ School of Life Science, Beijing Institute of Technology, Beijing, China; ^2^ College of Life Sciences & Research Center for Resource Peptide Drugs, Shaanxi Engineering & Technological Research Center for Conversation & Utilization of Regional Biological Resources, Yanan University, Yanan, China; ^3^ The Research and Development Center, China Resources Sanjiu Medical & Pharmaceutical Co., Ltd., Shenzhen, China

**Keywords:** oxidative stress, Zhengtian capsule, neurogenesis, brain-derived neurotrophic factor, GABAergic neurons

## Abstract

Oxidative stress that causes neural damages in neurodegenerative disorders has been widely studied for the pathogenesis and diagnostic measures. Zhengtian capsule (ZTC), a type of traditional Chinese medicine for headaches, has been found to have extra effects in recent years, such as promoting the release of serotonin and dopamine in the brain, but its specific mechanism has not been clearly elucidated. In this study, we focus on revealing whether ZTC can regulate key proteins of neurotrophic signaling pathway to alleviate depression-like behavior caused by oxidative stress. Experimental results show that ZTC (M 0.34 and H 0.7 g/kg) can elevate the proliferation of neural stem cells and GABAergic-type neurons in the hippocampus, promote the protein levels of BDNF, phosphorylated ERK1/2, and CREB, and inhibit the expression level of a key inflammation factor NFκB in a dose-dependent manner. These data suggest ZTC acts on multiple pathways to resist excessive oxidative stress, proving it to be a potential neurotrophic drug.

## Introduction

With the global acceleration of aging population, neurological diseases have raised to become the main killer of human health (Multhaup and Masters, 2018). Numerous studies reported that oxidative stress can cause lesions of nervous system and become a main pathogenic factor of many neurological diseases, such as Alzheimer disease, Parkinson’s disease (Islam, 2017; Poprac et al., 2017), and depression (Black et al., 2015; Lindqvist et al., 2017). This study focuses on explore whether the depression-like behavior caused by oxidative stress could be reduced with appropriate intervention.

Recently, the scientists have found that anxiety and depression-like behaviors can be alleviated by promoting neurogenesis in adult rats (Hill et al., 2015). It is reported that resveratrol, isolated from grapes, can promote neurogenesis and improve mood disorders (Kodali et al., 2015; Anacker et al., 2018). Studies of the role of resveratrol on the restoration of neural injury demonstrate that resveratrol can induce the expression of Nrf2, HO-1, and NQO1 and regulate the SHH signaling pathway to alleviate the damage and improve the proliferation of neural stem cells by oxygen-glucose deprivation/reoxygenation (Cheng et al., 2015; Shen et al., 2016). In addition, resveratrol is found to be able to improve age-related memory and mood dysfunction in mice through induction of neurogenesis and microvasculature and reduction of glial activation in aged mice (Kodali et al., 2015). Furthermore, resveratrol can also attenuate the LPS-induced depressive-like behaviors by induction of neurogenesis (Liu et al., 2016). Studies on oocytes found that resveratrol performs its pharmacological role in nervous system *via* modulation of the expression of GABAC receptors and its mediated ion-channels (Lee et al., 2013). Resveratrol also regulates cocaine-induced inhibitory synaptic plasticity of dopamine neurons in the ventral tegmental area by inhibiting phosphodiesterases (PDEs) (Li et al., 2017). Resveratrol can be well absorbed and fast metabolized in humans that about 75% of resveratrol can be diffused and absorbed through oral migration and its main metabolites are glucuronides and sulfates of resveratrol both in plasma and urine (Walle, 2011). Resveratrol is well tolerated and no significant toxicity has been reported yet (Cottart et al., 2010).

Structurally similar as resveratrol, Pterostilbene isolated from the Chinese herbal medicine Dragon’s blood can promote the development of adult rat neurogenesis, up-regulate BDNF (brain-derived neurotrophic factor, BDNF) and alleviate depression-like behaviors (Yang et al., 2019b). As being a traditional Chinese medicine, ZTC has been approved by the National Food & Drug administration Act (Cao et al., 2014). ZTC has been shown to play a role in migraines by improving blood circulation, reducing the damage caused by clotting, and inducing the expression of β-endorphins, serotonin, dopamine,and norepinephrine in the brain (Wu et al., 2014). Our recent studies have demonstrated that ZTC can alleviate the oxidative stress of Kunming mice. It is observed that, under the induction of lipopolysaccharide (LPS), administration of ZTC could down-regulate the levels of H_2_O_2_ and Malondialdehyde, and upregulate the levels of SOD and ATP in brain tissue, thus reducing the depression-like behavior caused by oxidative stress (Yang et al., 2019a).

Meanwhile, many similar studies also reported that upregulation of BDNF expression in brain tissue and promotion of neurogenesis in the hippocampus can alleviate mood disorders. Since the synthetic sites of BDNF in the central region of hippocampus are mainly in the CA1, CA3, and the hilus of dentate gyrus (Zakharenko, 2003; Lu et al., 2008), it is proposed that induction of the proliferation of endogenous neural stem cells and upregulation of BDNF may be a new strategy for the treatment of depression. Gamma-aminobutyric acid (GABA), an inhibitory neurotransmitter in the mammalian central nervous system, has been shown to be enriched in the resting-state of neural stem cells (NSCs) and hippocampal neurogenesis in the nervous system (Bao et al., 2017). The scientists found that hippocampal neural precursor cells in adult mouse begin with immature GABA synaptic input from small albumin expressing interneurons (Song et al., 2013).

In this study, we used a single injection of LPS to increase oxidative stress and established a depression-like behavioral model in mice. It was observed that ZTC could effectively alleviate depression-like behavior caused by oxidative stress and improve the exploration ability of stressed mice. ZTC enhanced the neurogenesis and GABAergic neurons in the hippocampus, and improved the expression levels of key proteins, such as BDNF, ERK1/2, CREB, but reduced the expression level of NFκB in the neurotrophic signaling pathway, which is consistent with previous studies (Bagul et al., 2015; Fang et al., 2016; Chen et al., 2018). This suggests that the role of ZTC reduction of LPS-induced inflammation is performed *via* regulation of NFκB related pathway. These data indicate that ZTC plays its role as a neurotrophic drug for depression treatment *via* induction of neurogenesis and regulation of neurotrophic signaling pathway.

## Methods

### Materials

Zhengtian Capsule (ZTC) (batch number: Z20010142) is a Chinese patent medicine, that is comprised of 15 traditional Chinese herbs: *Spatholobus suberectus* Dunn *(169.17 g/1 kg compound)*, *Angelica sinensis* (Oliv.) Diels *(56.06 g/1 kg compound)*, *Ligusticum chuanxiong* S.H.Qiu, Y.Q.Zeng, K.Y.Pan, Y.C.Tang & J.M.Xu *(101.10 g/1 kg compound)*, *Asarum caudigerellum* C.Y.Chen & C.S.Yang *(56.06 g/1 kg compound)*, *Uncaria rhynchophylla* (Miq.) Miq *(112.11 g/1 kg compound)*, *Paeonia lactiflora* Pall *(67.07 g/1 kg compound)*, *Rehmannia glutinosa* (Gaertn.) DC *(56.06 g/1 kg compound)*, *Angelica dahurica* (Hoffm.) Benth. & Hook.f. ex Franch. & Sav. *(56.06 g/1 kg compound)*, *Saposhnikovia divaricata* (Turcz. ex Ledeb.) Schischk *(56.06/1 kg compound)*, *Notopterygium incisum* K.C.Ting ex H.T.Chang *(56.06 g/1 kg compound)*, *Prunus persica* (L.) Batsch *(34.03 g/1 kg compound)*, *Carthamus tinctorius* L. *(34.03 g/1* *kg compound)*, *Angelica pubescens* Maxim *(34.03 g/1 kg compound)*, *Ephedra sinica* Stapf *(56.06 g/1 kg compound)*, and *Aconitum carmichaeli* Debeaux *(56.06 g/1 kg compound)* (The detailed information of these plants used in ZTC is listed in **Supplementary File**). These traditional Chinese herbs are supplied by the China Resources Sanjiu Medical & Pharmaceutical Co., Ltd. The complexity of ZTC has been analyzed by 2D-LC system that 876 peaks were detected and the peak capacity reached 1740 (please refer to the detailed information in literatures (Wei et al., 2009; Yang et al., 2019a). Resveratrol (RES, Sigma-Aldrich, USA).

### Animals

The study was performed in male Kunming (KM) mice (20–22 g) obtained from Animal Experimental Center of Peking University (Beijing, China, No SCXK (Jing) 2014-0013). Animals were housed under standard conditions (22–25°C, 12 h light/dark cycle) in cages. The mice were randomly divided into 6 groups (n=10 mice per group). After behavioral tests, mice were anesthetized with 60 mg/kg pentobarbital sodium (i.p), and then sacrificed for molecular experiment. When anesthesia, firstly, the mice were soothed and anesthetized by intraperitoneal injection; then the mice were thoroughly anesthetized and cardiac perfused to obtain brain tissue; later, the animal carcasses were handed over to the Beijing Experimental Animal Carcass Management Organization for unified processing. All experiments were carried out under the guidance of the Beijing animal ethics Association and the ethics committee of Beijing Institute of Technology (SYXK-BIT-school of life science-2017-M03).

### Drug Administration

ZTC and resveratrol were dissolved in water for intragastric administration at a volume of 0.1 ml per 10 g body weight. Different doses of ZTC were given continuously for 8 days *via* intragastric administration, and 5 mg/kg of LPS was administered intraperitoneally 1 h after the last day of administration. The mice were grouped based on the administration of ZTC dosages as follows: Normal group, 0.9% normal saline; model group (LPS), 5 mg/kg (Qin et al., 2013); positive group (resveratrol RES, Sigma-Aldrich, St. Louis, MO), 40 mg/kg, and the detailed administration of RES is described as: mice receive daily intraperitoneal injections of RES that is dissolved in 0.2 ml of 2% ethyl alcohol (Maheedhar et al., 2015); ZTC low dose group, (L) 0.17 g/kg; ZTC medium dose group, (M) 0.34 g/kg; ZTC high dose group, (H) 0.7 g/kg).

### Behavioral Testing

The behaviors of mice were tested 24 h after drug administration, including: the opening experiment and the balance experiment.

### Opening Experiment

Each mouse was placed in a 50 cm × 50 cm box, and the box was placed at a low square of 10 cm × 10 cm. In the test session, the numbers of crossing, rearing times and grooming times of the mice were recorded within 5 min.

### Rotarod Test

Motor ability was studied in KM mice utilizing a Rotary Fatigue Tester. During training, each mouse was placed on the rotating rod at a rate of 4 rpm for 3 min. Mice that were unable to hold onto the rod for more than 3 min were excluded from the study. In the test session, mice were place onto a rod, and the time durations of their latency to fall were measured.

### Immunofluorescence Staining

After continuous administration of ZTC for 8 days, BrdU was firstly injected twice by 2 h interval; and after 20 days, BrdU was again injected twice by 2 h interval; and after another 24 h, the mice were sacrificed and the brain slices were prepared for immunochemistry experiment. The mouse brain was treated with 4% paraformaldehyde for more than 48 h, and then dehydrated by two steps in 20% and 30% sucrose, separately. Coronal continuous sections, thickness 20 um, each 10 pieces were collected in a small hole in a 24-well plate. Subsequently, different samples were randomly sampled at the same location and stained and the results were counted (Liang et al., 2017). BrdU staining: Sections were blocked with 1% H_2_O_2_ in dark at room temperature for half an hour, and melted by 2M HCI at 37°C for 1 h, and the primary antibody (1: 500; Cat. No. MAB3424, Millipore) was incubated at 4°C overnight. GAD67 staining: brain sections were blocked with 0.5% Tween in PBS for 2 h, and diluted with 0.05% Tween in PBS for primary antibody (1: 500; Cat. No. MAB5406, Millipore) and incubated overnight at 4°C. DCX brain sections were blocked with 0.5% Triton-100 in PBS for 2 h, and diluted with 0.3% Triton-100 in PBS for primary antibody (1: 500; Cat. No. 4604, Cell Signaling) and incubated overnight at 4°C.

### Extraction of Total RNA and Quantitative Real-Time PCR (RT-qPCR)

Total RNA was extracted from the hippocampus of experimental mice, and the RNAiso Plus kit (Cat. No. 9109, TaKaRa) was used according to its kit protocol. cDNA extracted from the total RNA obtained in the previous step using a kit (Cat. No. RR036A, TaKaRa). The PCR step was as follows: 1 cycle at 95°C for 3 min; 39 cycles at 95°C for 10 s, and 60°C for 30 s (Song et al., 2018).

The primer sequences used were as follows: BDNF (F: 5’-GGACTCTGGAGAGCGTGAAT-3’; R: 5’-ACCTTCTGGTCCTCATCCAG-3’); GAD67 (F: 5’-CTCAGGCTGTATGTCAGATGTTC-3’; R: 5’- AAGCGAGTCACAGAGATTGGTC-3’); GAPDH (F: 5’-AAGGGCATCTTGGGCTACAC-3’; R: 5’-GGCCTCTCTTGCTCAGTGTC-3’). RT-qPCR was performed in SYBR^®^ Premix Ex Taq™ (Tli RNaseH Plus) (Cat. No. RR820A, TaKaRa), using Applied Biosystems’ StepOnePlus™ Real-Time PCR System. Data were analyzed using the delta-delta Ct method.

### Western Blot

The hippocampal tissues were lysed in RIPA buffer (Cat. No. R0010, Solarbio) containing the phosphatase and protease inhibitors. Protein concentrations were determined by the Bradford method. 10% or 12% SDS-PAGE gel were used to separate the proteins, and then which were transferred to the PVDF membrane (Cat. No. ISEQ00010, Solarbio). The membranes then incubated with primary antibodies diluted in TBST at 4°C overnight, then which were incubated with the HRP-conjugated secondary antibody at room temperature for 1.5–2 h (Liang et al., 2017). The primary antibodies used were: BDNF (1:1000, Cat. No. ab108319, Abcam), ERK1/2 or phospho-ERK1/2, CREB or phospho-CREB, NFκB or phospho-NFκB (1:2000, Cat. No. 4695, 9101, 4034, 4095, 3033, 3034, Cell Signaling), and the secondary antibodies used were: Goat anti Rabbit IgG (H+L)/HRP (1:5000, Cat. No. ZB2301, Zhongshan JinQiao biotechnology), Goat anti mouse IgG (H+L)/HRP (1:5000, Cat. No. ZB2305, Zhongshan JinQiao biotechnology).

### Statistical Analysis

Data were expressed as mean ± standard error of mean (SEM). The one-way analysis of variance (ANOVA) was used for data analysis. Difference associated with *P <*0.05 was considered statistically significant.

## Results

### ZTC Alleviates the Depression-Like Behaviors Induced by Oxidative Stress

**Figure 1:**
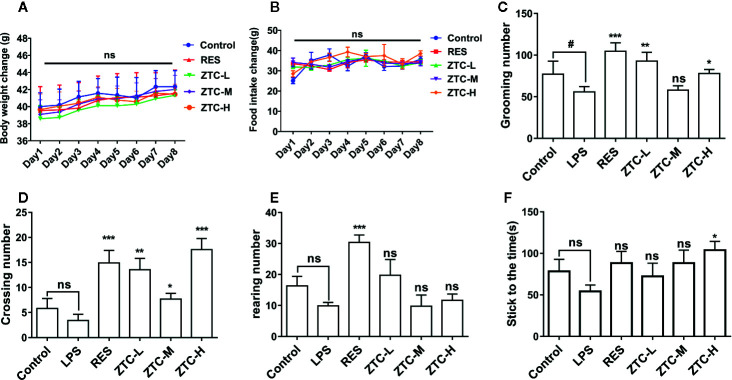
Behavioral tests and weight feeding tests of mice. **(A)** Changes of mouse weight for 8 consecutive days. **(B)** Detection of mouse food intake for 8 consecutive days. **(C–E)**. Open Field Experiment: the mice were divided into 6 groups, including control, LPS, RES, and ZTC (H 0.7 g/kg, M 0.34 g/kg, and L 0.17 g/kg). Numbers of grooming, crossing and rearing in 6 groups mice. **(F)** Balance test of 6 groups of mice. *p < 0.05, ^#^p < 0.05, **p < 0.01, ***p < 0.001 compared with stress group, by one-way ANOVA; ns, no significance; mean + S.E.M. in bar graphs.

ZTC was administered to mice in three dosage groups (H 0.7 g/kg, M 0.34 g/kg, and L 0.17 g/kg); resveratrol group (RES) (40 mg/kg, intraperitoneal injection) was used as a positive control (Kodali et al., 2015; Xu et al., 2015). The mice in the control group and model group were given the same amount of saline daily. We tested the weight gain of mouse and daily food intake of each cage mice. The result showed that different treatments had no significant effects on the body weight gain and daily food intake of mice (**Figures 1A, B**). The open field test was applied to assess animals’ ability to explore. Compared with the model group, the crossing numbers and the grooming numbers of ZTC-H and ZTC-L groups were significantly higher than that of the model group, though the rearing among those groups kept unchanged (**Figures 1C–E**), indicating that ZTC can effectively alleviate the depression-like state caused by oxidative stress and improve the exploration ability of mice under stress. The rotarod test was used to assess animals’ activity and balance. In the rotating test, the balance of the ZTC-H group was much higher than that of the model group (**Figure 1F**), suggesting that the high dose of ZTC can effectively improve the ability of coordination and balance of exercise of mice that are caused by oxidative stress.

### ZTC Induces Neurogenesis and GABAergic Neuron Numbers in Hippocampus

**Figure 2:**
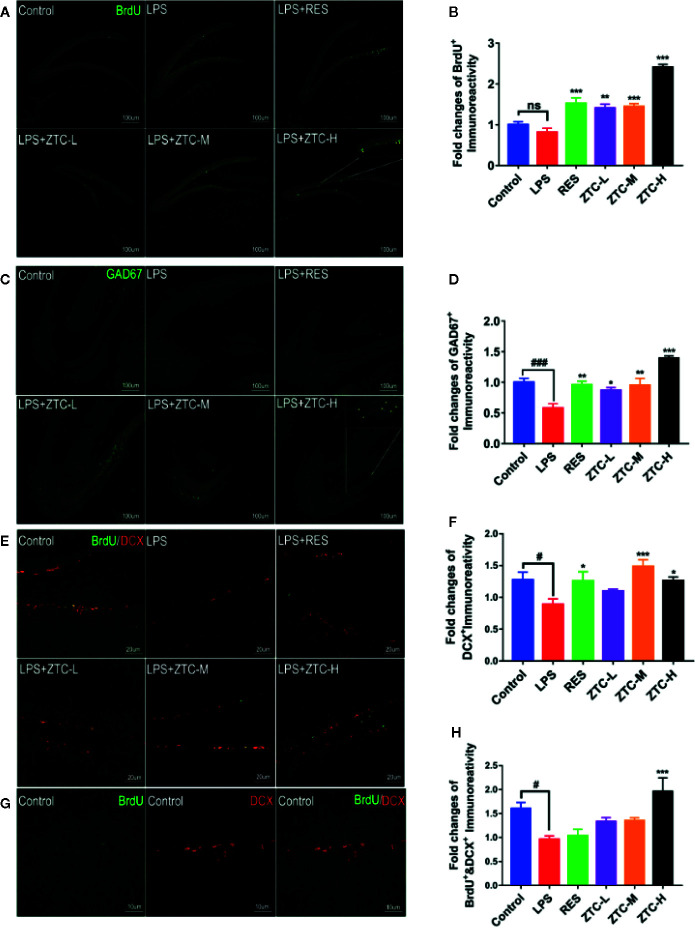
Neurogenesis and GABAergic Neuron Type Detection in Mouse Brain Slices. **(A)** Positive immunofluorescence staining of BrdU^+^-labeled cells in the hippocampal dentate gyrus of control, LPS, RES, and ZTC groups; (scale bar, 100 μm). **(B)** Fold changes of positive BrdU^+^ Immunoreactivity. **(C)** Positive immunofluorescence staining of GAD67^+^-labeled cells in the hippocampal dentate gyrus of control, LPS, RES, and ZTC groups; (scale bar, 100 μm). **(D)** Fold changes of positive GAD67^+^ immunoreactivity. **(E)** Positive immunofluorescence staining of BrdU^+^/DCX^+^ labeled cells in the hippocampal dentate gyrus of control, LPS, RES, and ZTC groups; (scale bar, 20 μm). **(F)** Fold changes of positive DCX^+^ Immunoreactivity. **(G)** Positive immunofluorescence staining of BrdU^+^-labeled cells in the hippocampal dentate gyrus of control group; (scale bar, 10 μm). **(H)** Fold changes of positive BrdU^+^/DCX^+^ Immunoreactivity. *p < 0.05, ^#^p<0.05, **p < 0.01, ***p < 0.001, ^###^p < 0.001 compared with stress group, by one-way ANOVA; ns, no significance; mean + S.E.M. in bar graphs.

Many studies have indicated that small molecule drugs such as resveratrol and shikonin can induce neurogenesis in the treatment of depression (Liu et al., 2016; Yang et al., 2019b). Therefore, we used immunohistochemistry to detect whether ZTC could induce the proliferation of hippocampal neural stem cells to improve hippocampal neurogenesis. The results showed that the number of BrdU^+^ labeled cells in the dentate gyrus of the hippocampus was significantly increased by ZTC treatment in a dose-dependent manner, compared to that by the LPS treatment (**Figures 2A, B**), indicating ZTC greatly induces the proliferation of neural stem cells. Fluorescence labeling was performed on hippocampal inhibitory neurons, histochemical staining showed that, after LPS treatment, GAD67^+^ labeled cells were significantly reduced compared with the control group, which could be reversed by RES treatment. Intriguingly, the ZTC-H mice produced much higher numbers of GABAergic neurons (GAD67^+^ labeled) in the hippocampal dentate gyrus than RES and other groups (**Figures 2C, D**). In addition, the co-immunostaining of DCX and BrdU experiments showed that LPS treatment reduced DCX^+^ positive neurons, while ZTC treatment resulted in increased DCX^+^ positive neurons (**Figures 2E–H**), suggesting that ZTC can restore the injury of hippocampal neural stem cells under oxidative stress. These data suggested that ZTC can resist oxidative stress at the tissue level by increasing the proliferation and migration of neural stem cells and inducing the number of GABAergic neurons in the hippocampus, which acts jointly on relieving the depression-like behavior caused by excessive oxidative stress (Ma et al., 2016; Duman et al., 2019).

### ZTC Recovers the Transcriptional Level of BDNF and GAD67 in Hippocampus

**Figure 3:**
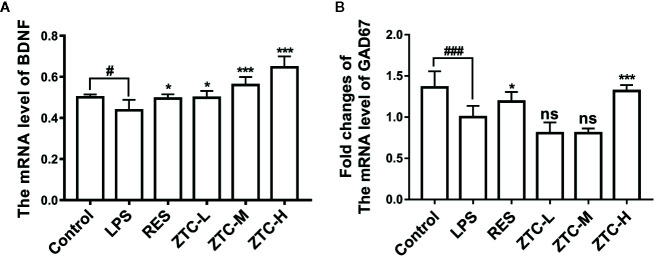
The mRNA expressions of BDNF and GAD67 in 6 groups of mice. **(A)** Detection of mRNA fold changes of BDNF in 6 groups of mice. **(B)** Detection of mRNA fold changes of GAD67 (that reflects the metabolic levels of GABAergic neurons) in 6 groups of mice. *p < 0.05, ^#^p < 0.05, p < 0.01, ***p < 0.001, ^###^p < 0.001 compared with stress group, by one-way ANOVA; mean + S.E.M. in bar graphs. ns, no significance.

The above results showed that ZTC can promote the proliferation of neural stem cells to improve neural damage caused by LPS, but its mechanism is unclear. Studies have shown that neurotrophic pathways have an important role in inducing proliferation of neural stem cells, in which BDNF acting as an important molecule is highly correlated with neurogenesis. GABAergic pathway also plays a leading role in the early stage of neural stem cell proliferation into neural cells. Therefore, we used RT-qPCR analysis to investigate whether ZTC treatment influenced the transcription levels of BDNF and GAD67 in the hippocampus. As shown in **Figure 3**, LPS treatment resulted in reduced mRNA levels of BDNF and GAD67 in the hippocampus of mice. ZTC can up-regulate the mRNA level of BDNF in dose-dependent manner. Meanwhile, the ZTC-H treatment significantly increased the transcriptional level of GAD67 in the hippocampus of mice (**Figures 3A, B**). These data implicated that ZTC regulates the neurogenesis *via* modulation of BDNF and GAD67 to alleviate neural damage caused by LPS.

### ZTC Affects Key Protein Levels of BDNF/ERK/CREB in Neurotrophic Signaling Pathway in Hippocampus

**Figure 4:**
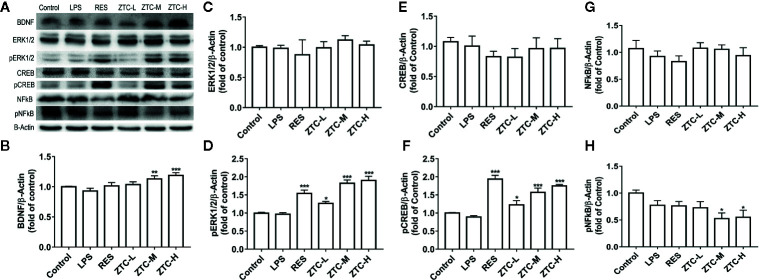
The expression levels of BDNF/ERK1/2/CREB/NFKB proteins in mice. **(A)** Representative Western blots showing BDNF, ERK1/2, CREB, NFκB, and β-Actin in the hippocampus of 6 groups of mice, respectively. **(B)** Relative immunoreactivity of BDNF normalized to β-Actin. **(C)** Relative immunoreactivity of ERK1/2 normalized to β-Actin. **(D)** Relative immunoreactivity of pERK1/2 normalized to β-Actin. **(E)** Relative immunoreactivity of CREB normalized to β-Actin. **(F)** Relative immunoreactivity of pCREB normalized to β-Actin. **(G)** Relative immunoreactivity of NFkB normalized to β-Actin. **(H)** Relative immunoreactivity of pNFkB normalized to β-Actin. *p < 0.05, **p < 0.01, ***p < 0.001 compared with stress group; mean + S.E.M. in bar graphs.

To further verify the involvement of neurotrophic pathway in the alleviation of depression-like behaviors by ZTC treatment, we also measured the related protein levels in neurotrophic pathway, including BDNF, ERK1/2, CREB, and NFκB. The western blot results showed that, the ZTC-M and ZTC-H groups significantly promoted the expression level of BDNF in the hippocampus in comparison to the LPS group (**Figures 4A, B**). Though the protein levels of ERK1/2 and CREB were kept unchanged, the phosphorylated proteins of pERK1/2 and pCREB in hippocampus were greatly improved by RES and ZTC treatments (**Figures 4C–F**). Similarly, the phosphorylated protein of pNFκB was much reduced by ZTC-M and ZTC-H treatments compared to that by the model group, but NFκB protein did not show any significance among groups (**Figures 4G, H**). Other studies reported that pERK1/2 and pCREB are responsible for the proliferation of neural stem cells (Yan et al., 2016; Choi et al., 2018); inhibition of the phosphorylation of NFκB can reduce the inflammatory effect (Ando et al., 2019). Thus, these data indicated that the increase of BDNF could induce the expression of other neurotrophic factors, and this might be motivated by induction of the proliferation of hippocampal neural stem cells and inhibitory GABAergic neurons, which further suppress the activation of NFκB and stimulate the phosphorylation of ERK1/2 and CREB. This process is considered as effectively antioxidative, thus reducing the oxidative stress.

## Discussion

Recently, botanic active components and their derivates have been prevalently studied for their roles in diseases and these studies focused on revealing the pharmacological mechanisms of botanic active components and their regulation in related signaling pathways (Fang et al., 2016; Teng et al., 2016; Teng et al., 2017b; Teng et al., 2017a; Chen et al., 2018). Agrimonolide from *Agrimonia pilosa*, exhibits a strong ability of anti-inflammation that is in part through inhibiting the activation of JNK and P38 MAPKs and decreasing the activation of JAK-STAT and NFκB in LPS stimulated macrophages (Chen et al., 2016). The dietary flavonoids can attenuate attenuating inflammation *via* targeting different tricellular signaling pathways triggered mainly by NFκB, AP-1, PPAR, Nrf2, and MAPKs, providing an insight for the development of new anti-inflammatory drugs in the future (Chen et al., 2018). This also inspired us to study the pharmacological effects and its molecular mechanism of ZTC in depressive-like behaviors under oxidative stress.

LPS modeling methods have been widely used in the establishment of stress models of nervous system in recent years, such as oxidative stress models, models of dopaminergic neuron degeneration induction, and models of inducing depression-like behaviors, and etc (Wei et al., 2009; Qin et al., 2013; Bagul et al., 2015). It is reported that single administration of LPS could lead to a decreased number of DCX^+^ positive neurons in WT mice and further induce neural injury (Valero et al., 2014). In our study, we applied single administration of LPS for establishing a short-term effect of depression-like behavior. This study, combined with our previous work, found that the administration of ZTC is basically safe and will not cause any adverse effects on the normal life of animals (Yang et al., 2019a). We continuously monitored weight gains and food intake in both experiments, and neither showed significant differences. Both the rotating rod and the open field tests indicated that ZTC could alleviate the depression-like behavioral symptoms of mice caused by oxidative stress, suggesting its anti-depression-like effects. The results showed that the treatment of ZTC can improve the ability of anti-pressure which can be a preventive strategy for depression.

Morphological results showed that ZTC could regulate the proliferation of neural stem cells in bilateral dentate gyrus of hippocampus and the expression level of GABA, the key factor of neurogenesis. The function of hippocampal neurogenesis varies from different regions. Dorsal dentate gyrus neurogenesis can promote learning and memory, while ventral neurogenesis can repair emotional disorders (Kheirbek et al., 2013; Weeden et al., 2015). GABA plays a leading role in the early proliferation of neural stem cell into neural cells. For the neurogenesis of mice, GABAergic transmitter inhibits the unintentional proliferation of neural stem cells, promoting the survived neural stem cells growing into neonatal nerve cells in the first 14 days, after 14 days, glutamine acidic neurotransmitters jointly induce neonatal neurons to become mature neurons that will integrate into neural networks (Kempermann et al., 2015; Toni and Schinder, 2015).

It is found that mice with higher neurogenesis show relatively lower depression symptoms and stronger social interactions compared with the control group (Song et al., 2018). Stress factors could reduce the incidence of adult neurogenesis. Decreased dentate gyrus neurogenesis caused by stress is an important factor contributing to the onset of depression. Interventional improvement of dentate gyrus neurogenesis during treatment will promote the improvement and recovery of depression (Hill et al., 2015). Neurogenesis is an important reason for the exertion of many antidepressants (Sapolsky, 2004). Adult hippocampal neurogenesis confers antidepressant effects by regulating dentate gyrus function. Studies also showed that ventral neurogenesis of dentate gyrus may be preferentially involved in mediating emotional disorders (Anacker and Hen, 2017). It is observed that the BDNF/ERK/CREB neurotrophic signaling pathway plays an important role in the improvement of depression-like behavior. The results showed that ZTC can significantly increase the transcriptional levels of BDNF and GAD67 regulatory genes in hippocampus of mice. These data indicated that ZTC may combat with the adverse effects caused by excessive oxidation in the body by activating the expression of key factors in BDNF-related neurotrophic pathway. Initiation of the transcriptional up-regulation of GABA is responsible for mood, exercise, learning and memory disorders (Creed et al., 2014; Qualls-Creekmore et al., 2017). Meanwhile, the protein levels of BDNF, pERK1/2 and pCREB were increased significantly under the intervention of ZTC. Studies have found that up-regulating the proliferation of neural stem cells in hippocampus can effectively improve the depression-like behaviors, and the mechanism of which may be related to the up-regulation of extracellular regulated protein kinase expression (ERK) (Chen et al., 2015; Nakka et al., 2016). Brain-derived neurotrophic factor (BDNF) plays a regulatory role in the proliferation and differentiation of neural stem cells. BDNF regulates the development direction of neural stem cells through signal transduction pathways such as MAPK kinase and cAMP protein kinase, and determines the type of differentiation of neural stem cells in the future (Islam et al., 2009; Zhang et al., 2018). The increase in the levels of these proteins and the directed proliferation of neural stem cells by ZTC play key roles in alleviating the depression-like behaviors of mice that are caused by oxidative stress.

## Conclusion

In conclusion, it is speculated that the induced expressions of BDNF, pERK1/2 and pCREB in the neurotrophic signaling pathway by ZTC treatment could help prepare nutrients for neural stem cell proliferation and reserve key regulatory factors for cell cycle. In addition, ZTC inhibits the activation of NFκB in mouse brain tissues that could promote the inflammatory response to reduce the oxidative stress *in vivo* caused by LPS. All these data indicated that ZTC’s anti-oxidative effect is synergistic through multiple pathways.

## Data Availability Statement 

The raw data supporting the conclusions of this article will be made available by the authors, without undue reservation, to any qualified researcher.

## Ethics Statement

The animal study was reviewed and approved by Beijing animal ethics Association and the ethics committee of Beijing Institute of Technology (SYXK-BIT-school of life science-2017-M03).

## Author Contributions

LY, NML, YW, BX, and CXY performed the experiments; ZZQ, LY, and QFYa analyzed the data; LY, NML, and ZZQ prepared the draft; JHD, ZD and QFYu helped data analysis; ZD, and HQ designed the experiments; ZD and ZZQ approved the final version. Both LY and YW contributed equally to this work. All authors have read and approved the manuscript.

## Funding

This study was funded by Shenzhen Collaborative Innovation Program (No. GJHS20140829143704130). At the same time, we had also received funding from other funds, the list was as follows: NSFC Regional Science Foundation Project (No. 31960174), and Yan’an University Research Project (No.YDQ2018-35, No. ydBK2019-14), Youth Program of National Natural Science Foundation of China (No. 81801073, 81701260).

## Conflict of Interest

Authors YW, BX, JD, QFYu, and ZD were employed by the company China Resources Sanjiu Medical & Pharmaceutical Co., Ltd.

The remaining authors declare that the research was conducted in the absence of any commercial or financial relationships that could be construed as a potential conflict of interest.

## Abbreviations

ZTC, Zhengtian Capsule; LPS, Lipopolysaccharide; GABA, Gamma-aminobutyric acid; BrdU, 5-Bromo-2-deoxyUridine; GAD67, Glutamate decarboxylase67; BDNF, Brain-derived neurotrophic factor; NFκB, nuclear factor kappa-B; DCX, Doublecortin Antibody.
