# Supplementary material for: The Anti-Depression-Like Effects of Zhengtian Capsule via Induction of Neurogenesis and the Neurotrophic Signaling Pathway
Source: Front Pharmacol. 2020 Aug 26;11:1338. doi: 10.3389/fphar.2020.01338 (PMC7479220; doi:10.3389/fphar.2020.01338)
Supplement: Supplementary file 1 [file DataSheet_1.pdf]

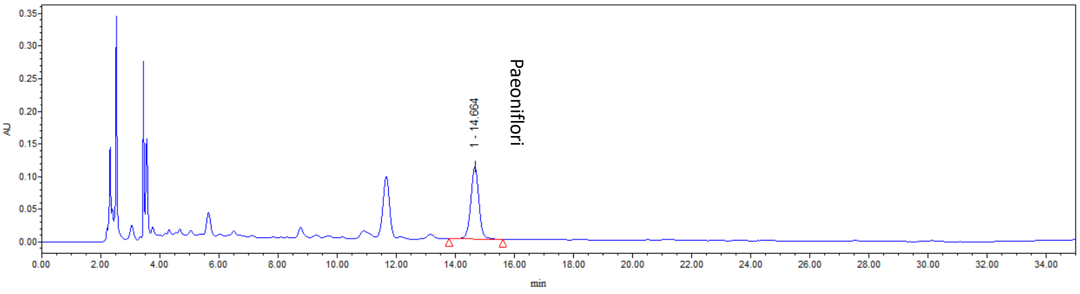

Figure 1.Chromatogram of the test product of Paeoniflorin of Zhengtian Capsule

The chromatogram shows the retention time of Paeoniflori is 14.664.

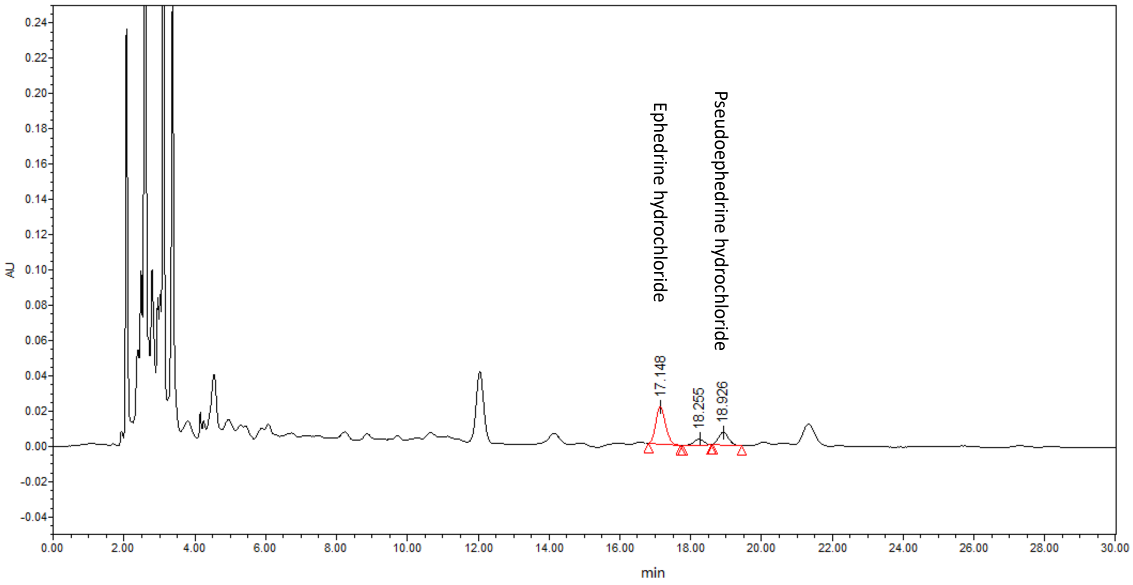

Figure 2.Chromatograms of Zhengtian capsule ephedrine hydrochloride and pseudoephedrine hydrochloride

The chromatogram shows the retention time of Ephedrine Hydrochloride is 17.148 and the Pseudoephedrine hydrochloride 18.926.
