# Supplementary material for: The Anti-Depression-Like Effects of Zhengtian Capsule via Induction of Neurogenesis and the Neurotrophic Signaling Pathway
Source: Front Pharmacol. 2020 Aug 26;11:1338. doi: 10.3389/fphar.2020.01338 (PMC7479220; doi:10.3389/fphar.2020.01338)
Supplement: Supplementary file 2 [file DataSheet_2.docx]

**Supplementary file：**

**The plants in Zhengtian Capsules（ZTC）and their parts for medicine：**

1. *Spatholobus suberectus* Dunn

<http://www.plantsoftheworldonline.org/taxon/urn:lsid:ipni.org:names:519185-1>

The medicine part is the dried lianoid stem

1. *Angelica sinensis* (Oliv.) Diels

<http://www.plantsoftheworldonline.org/taxon/urn:lsid:ipni.org:names:77065778-1>

The medicine part is the dried root

1. *Ligusticum chuanxiong* S.H.Qiu, Y.Q.Zeng, K.Y.Pan, Y.C.Tang & J.M.Xu

<http://www.plantsoftheworldonline.org/taxon/urn:lsid:ipni.org:names:844410-1>

The dried rhizomes are used as medicine

# Asarum caudigerellum C.Y.Chen & C.S.Yang

http://www.plantsoftheworldonline.org/taxon/urn:lsid:ipni.org:names:93505-1#sources

Dry roots and rhizomes are used as medicine

1. *Uncaria rhynchophylla* (Miq.) Miq.

<http://www.plantsoftheworldonline.org/taxon/urn:lsid:ipni.org:names:768297-1>

The dried hook-bearing branch is used as medicine

1. *Paeonia lactiflora* Pall

<http://www.plantsoftheworldonline.org/taxon/urn:lsid:ipni.org:names:711802-1>

The dry root is used as medicine

1. *Angelica dahurica* (Hoffm.) Benth. & Hook.f. ex Franch. & Sav.

<http://www.plantsoftheworldonline.org/taxon/urn:lsid:ipni.org:names:60451989-2>

The Dry root is used as medicine

1. *Rehmannia glutinosa* (Gaertn.) DC.

<http://www.plantsoftheworldonline.org/taxon/urn:lsid:ipni.org:names:808644-1>

The fresh or dried root tuber is used as medicine

1. *Saposhnikovia divaricata* (Turcz. ex Ledeb.) Schischk.

<http://www.plantsoftheworldonline.org/taxon/urn:lsid:ipni.org:names:847902-1>

The medicine part is the dried root

1. *Notopterygium incisum* K.C.Ting ex H.T.Chang

<http://www.plantsoftheworldonline.org/taxon/urn:lsid:ipni.org:names:845199-1>

The dried rhizome and root are used as medicine

1. *Prunus persica* (L.) Batsch

<http://www.plantsoftheworldonline.org/taxon/urn:lsid:ipni.org:names:1212858-2>

The medicine part is the dried ripe seed

1. *Carthamus tinctorius* L.

<http://www.plantsoftheworldonline.org/taxon/urn:lsid:ipni.org:names:324467-2>

The dried flowers are used as medicine

1. *Angelica pubescens* Maxim.

<http://www.plantsoftheworldonline.org/taxon/urn:lsid:ipni.org:names:837743-1>

The plant roots are used as medicine

1. *Ephedra sinica* Stapf

<http://www.plantsoftheworldonline.org/taxon/urn:lsid:ipni.org:names:383460-1>

The dried herbaceous stems used as medicine

1. *Aconitum carmichaeli* Debeaux

http://www.plantsoftheworldonline.org/taxon/urn:lsid:ipni.org:names:707241-1

The processed daughter root is used as medicine

**The taxonomy of plants in ZTC was rechecked and revised following the website provided by the editor and the 2020 Chinese Pharmacopoeia.**
